# Decreased motivation in the use of insecticide-treated nets in a malaria endemic area in Burkina Faso

**DOI:** 10.1186/1475-2875-8-175

**Published:** 2009-07-29

**Authors:** Léa Paré Toé, Olé Skovmand, Kounbobr Roch Dabiré, Abdoulaye Diabaté, Yveline Diallo, Tinga Robert Guiguemdé, Julien Marie Christian Doannio, Martin Akogbeto, Thierry Baldet, Marc-Eric Gruénais

**Affiliations:** 1 Institut de Recherche en Science de la Santé (IRSS)/Centre Muraz, BP 390, Bobo-Dioulasso, Burkina Faso; 2 UMR 912-SE4S, INSERM/IRD, Sciences économiques et sociales, systèmes de santé, sociétés, BP 13006 Marseille, France; 3 Intelligent Insect Control (IIC), 118 allée des Alouettes, 34170 Castelnau le lez, Montpellier, France; 4 Institut Pierre Richet/IRD 15 BP 917 Abidjan 15, Côte d'Ivoire; 5 IRD/CIRAD/Centre de Recherche Entomologique de Cotonou (CREC) BP06-2604, Cotonou, Bénin; 6 Centre de Recherche Entomologique de Cotonou (CREC) BP 06-2604, Cotonou, Bénin

## Abstract

**Background:**

The use of insecticide-treated nets (ITN) is an important tool in the Roll Back Malaria (RBM) strategy. For ITNs to be effective they need to be used correctly. Previous studies have shown that many factors, such as wealth, access to health care, education, ethnicity and gender, determine the ownership and use of ITNs. Some studies showed that free distribution and public awareness campaigns increased the rate of use. However, there have been no evaluations of the short- and long-term impact of such motivation campaigns. A study carried out in a malaria endemic area in south-western Burkina Faso indicated that this increased use declined after several months. The reasons were a combination of the community representation of malaria, the perception of the effectiveness and usefulness of ITNs and also the manner in which households are organized by day and by night.

**Methods:**

PermaNet 2.0^® ^and Olyset^® ^were distributed in 455 compounds at the beginning of the rainy season. The community was educated on the effectiveness of nets in reducing malaria and on how to use them. To assess motivation, qualitative tools were used: one hundred people were interviewed, two hundred houses were observed directly and two houses were monitored monthly throughout one year.

**Results:**

The motivation for the use of bednets decreased after less than a year. Inhabitants' conception of malaria and the inconvenience of using bednets in small houses were the major reasons. Acceptance that ITNs were useful in reducing malaria was moderated by the fact that mosquitoes were considered to be only one of several factors which caused malaria. The appropriate and routine use of ITNs was adversely affected by the functional organization of the houses, which changed as between day and night. Bednets were not used when the perceived benefits of reduction in mosquito nuisance and of malaria were considered not to be worth the inconvenience of daily use.

**Conclusion:**

In order to bridge the gap between possession and use of bednets, concerted efforts are required to change behaviour by providing accurate information, most particularly by convincing people that mosquitoes are the only source of malaria, whilst recognising that there are other diseases with similar symptoms, caused in other ways. The medical message must underline the seriousness of malaria and the presence of the malaria vector in the dry season as well as the wet, in order to encourage the use of bednets whenever transmission can occur. Communities would benefit from impregnated bednets and other vector control measures being better adapted to their homes, thus reducing the inconvenience of their use.

## Background

Several studies have shown that using an insecticide-treated net (ITN) is effective in reducing the man-vector contact and prevent malaria [1-3]. The use of ITNs on a large scale reduces clinical malaria episodes by 48% and saves 6% of 1,000 children below five years of age [4]. Despite the evidence that the use of ITNs decreases malaria-related morbidity and mortality, the use of ITNs in sub-Saharan Africa remains relatively low. Estimates from Africa as a whole indicated that, in 2005, only 3% of children less of than five years of age sleep under ITNs, while up to ten times as many are thought to sleep under any bednet[5]. Recent campaigns with free distribution of bednets have largely increased the ownership of ITNs [6], but some studies show that the incidence of use does not follow that of ownership and especially that the rate of use declines at the beginning of the dry season [7].

Notwithstanding the cost of ITNs on the open market in resource-poor countries, several studies carried out in English-speaking African countries have attributed the low usage to practical and technical difficulties related to the fixing of the net above the mat, the design of the house [5,8], the feeling of suffocation and discomfort related to the relatively high temperatures even during the night, and the preferred use of local methods against mosquitoes, such as herbal repellents [8,9]. Socio-economic factors, such as wealth, access to health care and education have been shown to be important predictors of ITN ownership and use [10,11], although the relative importance of purchase power declines where bednets are distributed for free or are highly subsidised [11]. Ethnicity has also been reported as an important factor, as people with pastoralist and semi-nomadic lifestyles may be less likely to own and use an ITN, as compared to settled agricultural communities [12-14]. Some research suggests that gender may influence the use of ITNs within households, as different roles dictate different sleeping patterns for men and women. Additionally, it seems that the main purpose of bednet usage is to protect against mosquito bites rather than to prevent malaria [8,9,15,16].

The underlying belief in this attitude often undermines the consistent use of bednets during the dry season, when mosquitoes are less noticeable [17-19]. However, nets should be used year-round in malaria endemic regions of sub-Saharan Africa, because there can be a substantial risk of transmission even when the vector density is low [20,21].

The lack of availability, and failure in the distribution systems of, ITNs have been identified by Roll Back Malaria (RBM) as the main limitations (other than cost) of large-scale implementation of ITN use [10,22]. One issue is whether the free distribution or subsidised sale of the nets will increase their usage. Proponents of free distribution emphasize the urgency for immediate results, whereas those who favour a more pluralistic approach, including the development of domestic markets for ITNs, are keen to ensure the long-term sustainability of delivery [12,23-26]. While this question is important and requires the need for multiple strategies in order to achieve and sustain high coverage rates, especially for the more vulnerable groups [27,28], it is crucial to understand what are the main motivations of any given populations for their use of ITNs.

Baume and Marin [29] mentioned some cases of non-use of nets during the rainy season and explained it in part by the desire to use new nets rather than old nets. This poses a problem for net usage in the long term. Whether the net is free or for sale, it is the perception of the individual that determines its consistent use. If the individuals are not sufficiently motivated to use ITNs in their daily life, their widespread use on a long-term basis will not be successful. Preliminary studies in a rural area in Burkina Faso revealed that the population used ITNs at high rates for the first few months and then gave it up for non-objective reasons, such as the reduction in the number of mosquitoes, difficulty in fixing the nets etc. Therefore, understanding what determines a reduced adherence to use is necessary in order to encourage and to maintain the use of ITNs in the long term.

The aim of this study was to: (i) study the social representation of malaria in a rural endemic area of sub-Saharan Africa; (ii) determine the perceived utility of ITNs within this context; (iii) study the functional and temporal organization of households; and (iv) specify how the social representation of malaria and ITNs associated with the design of the house can limit the use of ITNs and, thus, hinder a long-term malaria control programme.

The final objective of this analysis was to examine the influences on the possession and use of ITNs and to extract lessons applicable to the West African context, or more broadly to other programmes that use ITNs, in order to reduce the burden of malaria.

## Methods

The study was carried out in Soumousso (11°00'46"N, 4°02'45"W), a village located in the humid savannah of south-western Burkina Faso. Soumousso is a typical Guinean savannah village situated about 55 km east from Bobo-Dioulasso, the second largest town in Burkina Faso. Three main malaria vectors, *Anopheles gambiae *(M and S form), *Anopheles funestus *and *Anopheles nili*, are found in this village. *Anopheles arabiensis *is occasionally reported at low frequency, reaching 5% of *An. gambiae *s.l. samples. The sporozoïte rate was about 3.6% and 2.9%, respectively for *An. gambiae *and *An. funestus *with an annual entomological inoculation rate (EIR) reaching 500 infected bites per person [30].

There are two distinct seasons in this village: rains occur from May to October followed by a long, dry season from November to April. The average annual rainfall ranges from 1,000–1,200 mm (records of the past five years). In Soumousso, malaria is qualified as holoendemic, typical of the tropical rural regions of humid savannah in western Africa. The transmission season is long (over six months) and peaks in October/November, at the end of the rainy season. In the study area, malaria remains the most common reason for attending a health facility. The population is composed of different ethnics groups: Dioula, Bobo, Mossi, Samo and Peulh, and the principal language is Dioula.

Most houses in Soumousso have a single common room and are rectangular or circular and made of mud, including the roofs. They have one door and some have no windows. They generally are organized in compounds, maybe several houses per compound. The sleeping arrangement is collective. Soumousso village has 455 compounds.

Insecticide-treated nets, including PermaNet 2.0^® ^and Olyset^®^, the long-lasting insecticide-treated bednets (LLINs) approved by the WHO Pesticide Evaluation Scheme, were distributed free-of-charge, in order to evaluate and compare their acceptability and to identify the criteria for their acceptability. They were given to the heads of the family as well as to their wives, both to investigate the perceptions of both sexes and to avoid husbands taking their wives' nets, if the latter were the only recipients. The ITNs were distributed at the beginning of the rainy season by the research team, after a meeting with the community leaders and the sensitization of the community to the objectives of the project, bednet usefulness and the net hanging process. All 455 compounds were covered. Medical staff explained about malaria, its transmission and the role of mosquitoes and emphasized the fact that malaria could be lethal.

The study lasted three years. During the rainy season in the first year, one in three persons had stopped using the net. The initial willingness to use the nets at the time of distribution was not maintained twelve months later. Therefore, the study was enlarged in order to discover the reasons for this reduced motivation. This paper presents the findings for this decreased motivation for net use during the rainy season.

Qualitative tools were required so as fully to understand the determinants for net use or non-use. Formal, in-depth discussions with household members, direct observations and photography were used.

The in-depth discussions allowed us to investigate the community perception of malaria and the reasons underlying the steps taken towards its prevention, as practised. It also enabled an understanding of the link between the community representation of malaria and the biomedical usefulness of ITNs. 100 men and women between the ages of 15 and 60 years were interviewed following a formal protocol, to supplement the information concerning decreased motivation for bednet use collected during the informal monthly visits to all inhabitants. 50 were chosen semi-randomly from net users and 50 from net non-users, by taking every third name on the village list of inhabitants. The objectives and method of the study were explained in the local language, after which informed consent was obtained from all participants in the study.

Two hundred houses were included in the direct observations. Soumousso village included 8 districts. 25 houses were chosen randomly per district from a list of village houses by picking every second household from the village list of households. The temporal and functional organization of the household, the sleeping arrangements and practical conditions of ITN use (hang, spread) were observed by following the arrangement of the space of the houses day and night. Finally, two households representing the basic models of a single-roomed house and a double-roomed house were followed monthly for a year in order to understand how the space inside the houses was used within the community. The purpose was to see if the management of space and of the net inside this space was related to the availability of space or to different sections of the domestic space.

Direct observations were made on, and informal discussions were conducted with, one or more members of the household. Photographs were taken to judge the spatial arrangement of the interior of the houses and the conditions under which the ITNs were used in two recurrent models, i.e. single- and double-roomed houses.

## Results

### Malaria as several nosological entities

The study population used many terms to translate the medical word "malaria". In the social representation, this medical word referred to many diseases. *Sumaya *(coolness), *sumaya gwe (*coolness white), *djakadjio *(malaria which has been in the body for a long period of time), were the words commonly used. However, *sumaya *was the most commonly used word to describe malaria. These diseases had some common symptoms, such as "hot body, headaches." Some respondents used *sumaya *interchangeably with '*palu*,' which is a diminutive of the French term "paludisme," to indicate malaria. It is difficult to make a clear differentiation between these diseases, since they have many symptoms in common. Accordingly, the local representation of "malaria" is inconsistent.

### Malaria, an endemic and ordinary sickness

The surveyed population declared that malaria occurs throughout the year and intensifies during the rainy season.

"*We have malaria all days, you cannot spend one day without somebody saying that he has malaria*" (a 30 year old man).

This endemic nature of malaria made it an ordinary sickness known by every household member: "*everybody knows sumaya here, because everybody has already got sumaya" *(a 35 year old woman). This "malaria familiarity" leads the population to consider malaria merely as a common disease and not a cause for concern: *"when you have a sumaya and you take medical or traditional medicines, the disease is finished. Sumaya is rarely serious" *(a 40 year old woman). The mother of a child with a headache and fever said: "*He has *sumaya. *The sickness began yesterday. He did not sleep at night. I gave him a plant concoction. He will get cured, sumaya is not serious*. "She left the house many times, leaving the child in the care of his 8 year old brother. Treatment at home without a referral to the dispensary, was common, since all householders knew the treatment and often treated themselves without outside help: *"It is not difficult to treat malaria, you only must begin early; when a child has a headache or *a sumaya, *I give him a concoction of plants or a quinine drug purchased from the pharmacy"*

### Community representation of malaria versus the biomedical usefulness of ITNs

Public health practitioners have presented the use of ITNs as the best strategy to reduce contact between man and mosquito, the sole cause of malaria transmission in the scientific definition. In the present study, the majority of respondents identified mosquito bites as one cause of malaria, but most of them named additional causes as well: "*When the weather is cold, the *sumaya *comes; when the mosquitoes bite you, you also get *sumaya" (a 40-year old man). "*We often have *sumaya *because we sleep in the coolness and there are many mosquitoes*" (a 45 year old mother). Some people clearly mentioned the type of mosquitoes which are responsible for malaria. For instance, the statement of a young interviewee of around 19 years of age: "*Malaria is transmitted by An. gambiae*". Those who gave the vector's name had been to school for a few years.

Various types of food were also identified by the population as a cause of malaria. One interviewee who was sick said "*I have *sumaya. *Last week I visited our farmer and drank a lot of milk, I know why I have *sumaya, *because the milk was very sweet and it gave me malaria" *(a 35 year old woman).

### Bednet usefulness according to the population

Several studies, including this one, identified that the ITN is initially used by a population to avoid mosquito bites. The population used the ITN when they "*felt disturbed*" or "*saw*" and heard the mosquitoes. When they were not bothered by the mosquitoes, they did not use the ITN, even in the high transmission period. Thus, some people interviewed in October-November at the peak of malaria transmission said *"I am not sleeping under my net because for the moment, there are not enough mosquitoes"*.

That interviewees had a low motivation for using bednets was illustrated by comments such as "*I forgot*" or "*I will do it tomorrow*" and by the use of damaged nets, even where the family was financially and technically able repaired or changed the nets: "*We did not think of it, we will do it later."*

In order better to understand this low motivation, interviewees were asked for the real reasons for the non-use of their nets. One 35 year old woman said "*Why do you want us to use the bednet always, when it does not protect us completely from malaria? The bednet does not protect against the cold, which also gives malaria. The bednet does not protect us enough against malaria. It helps us to sleep well; we will make an effort to fix it the day there are many mosquitoes"*.

### Instructions on using the bednet are at odds with the normal interior functional and temporal organization of the house

**Figure 1:**
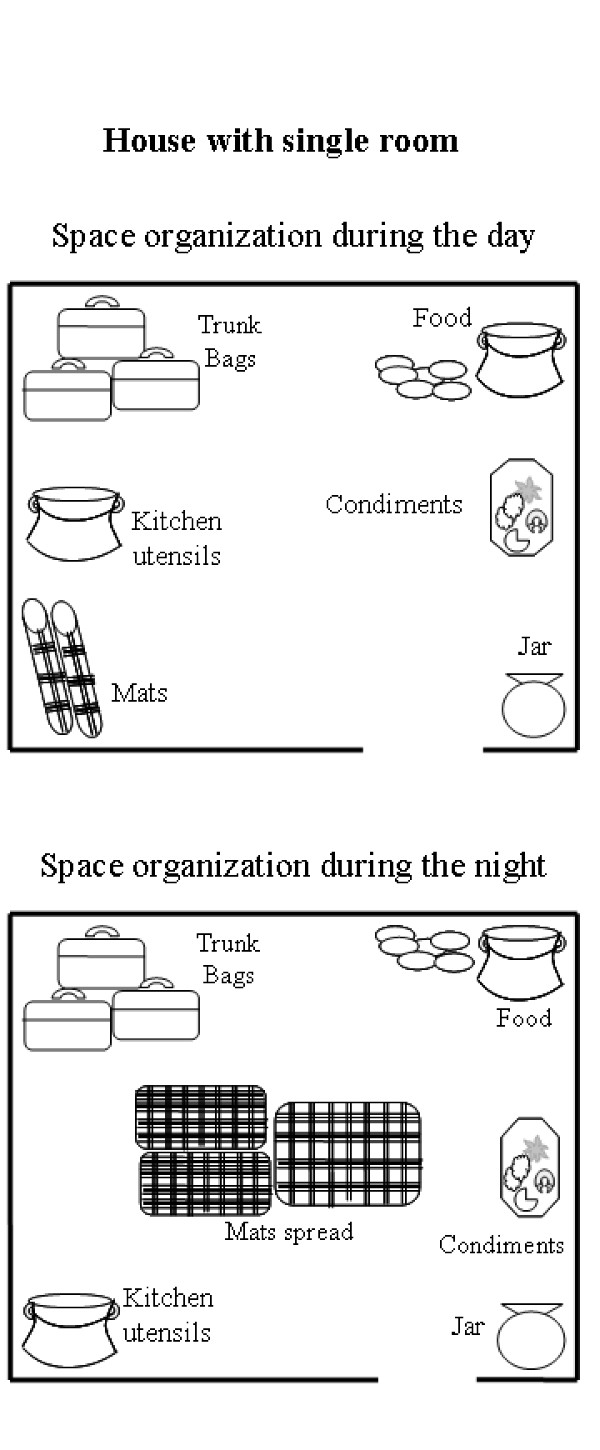
**House with single room**.

The bednet is a physical object that takes up a relatively large space. The use or non-use of any object depends on the interest the user has in it, its perceived usefulness and the problem it causes by its physical presence and by its daily removal and redeployment. On the perception of the interviewees, the bednet did not totally play all the roles that promote its use. It reduced mosquito nuisance, but it was not perceived as removing all causes of malaria. Bednets must be fixed indoors where the inhabitants sleep and the management of the interior of a house differs between daytime and night-time. Below are two examples of the inside management of two houses during the day and the night. The two examples illustrate the functional and temporal organization of single- and double- roomed houses (Figure 1).

**Figure 2:**
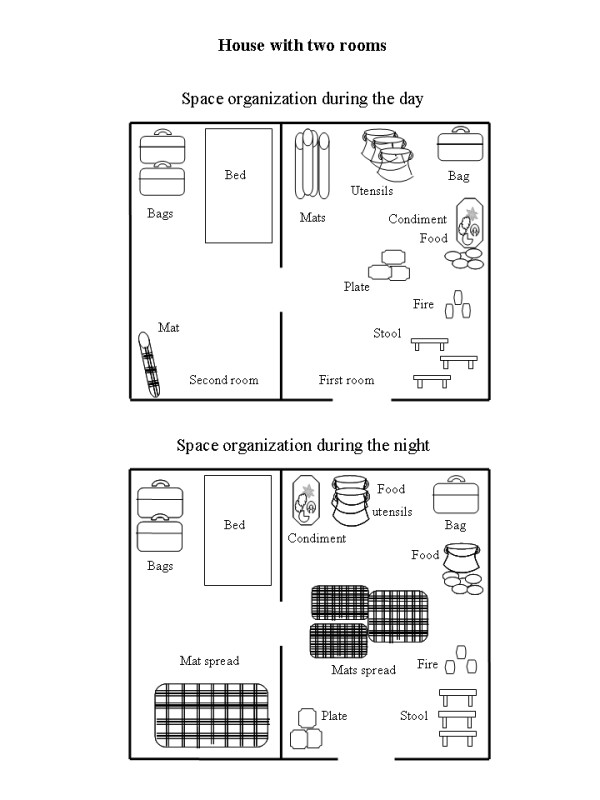
**House with two rooms**.

During the daytime in the single-roomed house, three mats were folded and placed in one corner and a jar of water in the opposite corner. Kitchen utensils, food, and dry and fresh condiments were spread out. A fire was built within and used for cooking when it rained. The inhabitants had their meals in this room. At night, the moveable items were stacked in the corners and the mats spread out in the centre of the room. As illustrated, the materials were stored close to the walls, making space for sleeping (Figure 2). Mats were spread on the floor for sleeping and used by the mother and her three sons aged between 4 and 13 years.

In the double-roomed house, the rooms were separated by a small wall. Seven people lived in this house, including the mother (about 50 years of age), her daughter and son aged 20 and 30, respectively, her two nieces of 10, and two grandsons of 8 years and of 9 months. The mother, one son and one grandson slept in the first room. The other four slept in the second room. In order to tidy the first room, the same method was followed as in the single-roomed house: the material less used at night was stored along the walls. In the second room, the trunks containing clothes, plates and many objects rarely used and the bed remained in place. The two mats were spread out close to the bed.

Those sleeping in these two houses did not use the mats at random. The bed management followed a rule. In the first house, as in the second, the mothers slept with their small sons; the young men slept together. Young girls always slept in their mother's room even if there was another empty room. A household member explained these sleeping arrangements as follows: "*the young girls must sleep in their mother's room, because the latter must keep an eye on their nocturnal outings*". Clearly, dedicating bednets to some and not others may disturb the sleeping patterns, and the daily use of bednets in these rooms is not easy. Moreover, the suspended nets may catch fire in the course of cooking.

## Discussion

Although ITNs were given free to the population and education on their proper use was addressed, especially to women during the net distribution, not everyone slept under an ITN every night. Although most people have the capacity to protect themselves, not everyone does so on a nightly basis. It is emphasized that this survey was conducted during the second half of the rainy season, at the peak of malaria transmission and, therefore, net usage should have been at its maximum.

It is possible that the initial high acceptance rate was related to the adoption and spread of a new technology, whereby people believed it was enough to be an 'ITN owner' and accepted a free net because they were offered it, rather than because they planned to use it or thought that they needed it.

Baume and Marin [29] showed that nets distributed free were less used than nets purchased. It has been suggested that this difference can be reduced by the use of information campaigns. In the present study, the reduced use of nets took place despite nets being distributed during an intense campaign of sensitization. No interviewee alluded to its free distribution as a reason for less use. The main reasons for non-use of ITNs can be explained by the community perception of malaria which results in, on the one hand, a limited perceived usefulness of ITNs and on the other hand the obvious problem of having a bulky product suspended in the middle of a room used for many purposes other than sleeping. Below, the impact the concept of malaria and its origin and of the limited space in the houses has on the acceptance of the daily use of bednets is considered, as are the problems in having a net (bulky and a possible fire risk) hanging in rooms which change function from day to night and where people cannot be allocated space together in order to let most vulnerable sleep under bednets, because sleeping patterns follow social rules.

Malaria was not described as a single, nosological entity, as also shown by previous studies carried out in Ghana, Tanzania and the Ivory Coast [13,14,17,31,32]. The social perception of the medical term "malaria" referred to many diseases, such as *sumaya, sumaya guè *and *djakadjo*. The description of these diseases showed that there are some common features in the causes and the symptoms, but that they did not necessarily refer strictly to the same disease. The different symptoms of these three diseases coincided with the different stages of malaria according to the medical definitions: "simple" and "severe" malaria [31], but may also include other diseases, such as bacteraemia. Importantly, severe malaria with convulsions is often seen as a disease apart which calls for different treatment [31].

Since "malaria" is not a unique reality for this population, it is logical that they deduce that this disease complex is not acquired solely from mosquitoes. Accordingly, they do not consider ITNs a total defence. Therefore it is necessary to create a setting for discussion between the medical staff and the population on the cause and symptoms of malaria and the advantages of using ITNs. Adongo *et al*[17] and Essé *et al *[31] discussed the incorporation of local terminology and the medical definition of malaria. Increased knowledge of malaria could lead to the synchronization of local and biomedical knowledge in the long term, meaning that people would understand that "palu" is a single entity, although similar symptoms can be due other diseases.

The inaccurate perception of malaria was based on the endemic character of malaria, which made it an "*ordinary disease*" [33], not perceived in the same way as an epidemic disease, such as meningitis, which, at times, kills many people in the same areas in Burkina Faso [34]. Such epidemics frighten people due to the great number of deaths which they attribute to the disease. This characterisation of malaria makes it accepted as a fact of life – a phenomenon unworthy of particular attention – and hence people do not always take the necessary precautions to prevent it.

Previous studies in East Africa revealed that the incidence of malaria and immunity levels within a population could influence community perceptions of the severity of the disease and might influence the perceived importance of taking precautions, such as bednet use [35,36]. Some studies [35,36] show that vulnerable groups (young children and women of reproductive age) sleep under nets more than others adults, indicating that when the method of prevention is already available within the household, priority is given to the more vulnerable. Others in the household did not show any particular interest in the method of prevention.

The medical programmes conducted in the villages informed the population that malaria is a lethal disease. However, the population did not see it as such. Even when the medical staff declared that death was due to malaria, the population sometimes attributed it to other causes. Despite the seriousness of the health problem, it is its social perception and beliefs that determine peoples' behaviour [16,37]. All these aspects result in a situation where malaria is not considered a serious disease that could be prevented by the use of ITNs. All the same, some people invest in protection against mosquitoes[33], but to fight against nuisance rather than disease.

In order to prevent a disease, the cause must be recognized and avoided. In practice, malaria protection consists of avoiding the different causes perceived by the people themselves. They prevent their children from going out in the rain and eating sweet and fatty foods and sometimes they organise the children to sleep under ITNs. Attempting to prevent malaria by the use of all these practices, rather than only using ITNs, was conceived as logical, because mosquitoes were not perceived as the only cause of malaria [2,9]. Therefore, this did not allow the population to adhere strictly to the public health programme message on the use of ITNs (seen as a daily complication of life) as an excellent tool for malaria prevention. The acceptability of physical objects depends on objective criteria, which are appearance, usefulness, and instruction on their use (easy/difficult) [18,38]. The object must be found to be practical and judged to be useful by the user.

The inhabitants in the study area found the design of the ITN positive and pleasant. Initially they wanted to acquire ITNs, but later the motivation for their use decreased and their usefulness was questioned.

The study describes a functional and temporal organization of house space, where its management differed between daytime and night-time. In daytime, the house inhabitants were outdoors, the commonly used objects were also outside and houses were relatively empty. Sleeping mats were stored away. At night, the space within was much solicited. The mats were spread close to different objects, including the fire place. The inner space of a house, including the sleeping area, assumed many functions. Many objects were stored and many activities carried out in the same area. The insertion of bednets in such a space was not easy for the inhabitant because, once set up, the bednets are fixed, limiting the sleeping arrangements and any other use of a significant part of the house. Further, it was difficult for them to erect the nets and take them down daily. Similar studies carried out in Kenya [5,8] also showed how the practical conditions of bednet use can limit their acceptability.

Difficulties in the use of the bednets were more related to space management than to the form of the houses (rectangular or circular). Space management was easier in double-roomed houses than in single-roomed houses. The majority of those who continually used their bednets had at least two rooms and had a less usual management of space, having dedicated sleeping places. Aside from such space management, the nuisance value of mosquitoes headed the reasons for the use of bednets, not just directly for protection against bites: some mothers revealed that they used bednets in order to avoid their children crying at night as a result of mosquito bites.

This study was limited by lack of quantitative data that would give the percentage decrease in net use motivation in vulnerable groups and the socio-demographics characteristic of non-users as found in other studies carried out in East Africa [8,39]. The qualitative, ethnographic approach was chosen to complement quantitative studies in order better to explain the reasons for the decreased motivation, based on the local population's daily lives.

In addition to the difficulties related to the cost and accessibility of bednets, improvement of the rate of usage requires much better communication between health professionals and social scientists and local populations on the causes of malaria, its severity, more particularly for those most vulnerable (pregnant women and children less than five years of age), and efficient prevention strategies. To create and sustain a strong motivation for ITN use, year-round, targeted and specific education campaigns must be adapted to the beliefs and behaviours that already exist in local communities, correct false assumptions and introduce distinctions. It is not helpful, as often found [31,40,41], that nurses and doctors routinely treat malaria-like symptoms as malaria without proper diagnosis, thus compounding ancient disease concepts. Such improvements are necessary, both in the short and the long term, if the ITN strategy advocated by RBM is to become truly effective.

## Conclusion

This study appears to be the first to evaluate the motivation of the use of bednet several months after free distribution combined with an intense motivation campaign. A decrease of motivation was noted during the first year of bednet use, despite the intense campaign that initially led to a high user acceptance and use in the first months. This raises the question of how well adapted general campaigns with bednets are, when the campaign does not address the disease concept and the physical frames for the use of ITNs. In the present study, the medical message as to the seriousness of malaria often did not convince the population and therefore the consistent use of bednets did not become a priority. To improve malaria prevention, it is necessary to align the population's understanding of malaria more closely with its medical reality and to make the ITN more practical to use, especially in small houses. Sleeping patterns follow social rules. Groups of children often sleep together, and these groups may not fit well into the sizes of bednets routinely distributed. The study shows that the sustained use of bednets is much more complex than most campaigns plan for.

## Abbreviations

EIR: entomological inoculation rate; ITN: insecticide-treated nets; LLIN: Long-lasting insecticide-treated bednets; RBM: Roll Back Malaria; WHO: World Health Organization.

## Competing interests

The authors declare that they have no competing interests.

## Authors' contributions

LPT participated to the study design, undertook the field study, analysed data and wrote the paper. YD, MEG, JMCD and MA participated in the analysis and interpretation of data. KRD, AD, OS, TB participated in the study design and the drafting and revision of the manuscript. TRG is the administrative authority who facilitated the implementation of the study. All authors have read and approved the final manuscript.
